# Lactate facilitated mitochondrial fission-derived ROS to promote pulmonary fibrosis via ERK/DRP-1 signaling

**DOI:** 10.1186/s12967-024-05289-2

**Published:** 2024-05-21

**Authors:** Zhiheng Sun, Zhihua Ji, Huiwen Meng, Wanyu He, Bin Li, Xiaoyue Pan, Yanlin Zhou, Guoying Yu

**Affiliations:** 1 College of Life Science, Institute of Biomedical Science, Henan Normal University, Xinxiang, Henan China; 2 State Key Laboratory of Cell Differentiation and Regulation, Henan, China

**Keywords:** Idiopathic pulmonary fibrosis, Reactive oxygen species, DRP1, Lactate, Mitochondrial fission

## Abstract

Idiopathic pulmonary fibrosis (IPF) is a chronic, progressive, fibrotic interstitial lung diseases, which mainly existed in middle-aged and elderly people. The accumulation of reactive oxygen species (ROS) is a common characteristic of IPF. Previous research also shown that lactate levels can be abnormally elevated in IPF patients. Emerging evidence suggested a relationship between lactate and ROS in IPF which needs further elucidation. In this article, we utilized a mouse model of BLM-induced pulmonary fibrosis to detect alterations in ROS levels and other indicators associated with fibrosis. Lactate could induce mitochondrial fragmentation by modulating expression and activity of DRP1 and ERK. Moreover, Increased ROS promoted P65 translocation into nucleus, leading to expression of lung fibrotic markers. Finally, Ulixertinib, Mdivi-1 and Mito-TEMPO, which were inhibitor activity of ERK, DRP1 and mtROS, respectively, could effectively prevented mitochondrial damage and production of ROS and eventually alleviate pulmonary fibrosis. Taken together, these findings suggested that lactate could promote lung fibrosis by increasing mitochondrial fission-derived ROS via ERK/DRP1 signaling, which may provide novel therapeutic solutions for IPF.

**Supplementary Information:**

The online version contains supplementary material available at 10.1186/s12967-024-05289-2.

## Introduction

Idiopathic pulmonary fibrosis (IPF) is a chronic, progressive and fibrotic lung disease with an unknown cause and insidious onset and lack of effective treatment [1, 2]. The median survival rate of IPF is only 2 ~ 5 years, which seriously affects life quality of patients with high mortality rate [3]. During normal lung injury repair, activated interstitial fibroblasts/myofibroblasts deposit extracellular matrix (ECM) composed mainly of fibrillar collagen and fibronectin, forming a temporary matrix that promotes proliferation and differentiation of type 2 alveolar epithelial cells (AEC2) progenitors to replenish damaged and detached epithelium [4]. Abnormal proliferation of myofibroblasts lead to increased stiffness and altered mechanical properties of lung tissue [5], which released profibrotic and proangiogenic signals [6]. Further understanding of specific pathogenesis mechanism of IPF is important in finding more rational treatment modalities [7].

Lactate accumulation in IPF was observed due to the prominence of metabolic disorders and mitochondrial dysfunction [8–10]. Moreover, mitochondrial network fragmentation led to a decrease in mitochondrial membrane potential and an elevation in mitochondrial reactive oxygen species (ROS) levels [11]. ROS were produced through the partial reduction of molecular oxygen (O2) and subsequent reactions with other molecules [12]. The primary sources of ROS in mammalian cells included mitochondrial electron transport chain, NOX enzymes, and production of hydrogen peroxide (H_2_O_2_) [13]. ROS could contribute to progression of pulmonary fibrosis through various mechanisms, including inflammation, oxidative stress, and alterations in glucose metabolism [14, 15]. However, the relationship between lactate and ROS in pulmonary fibrosis had not been completely elucidated.

DRP1 (dynamin-related protein 1, also known as DNM1L) is the dynamin superfamily of GTPases, which was involved in modulating mitochondrial morphology [16]. Phosphorylation was one of the most well-studied post-translational modifications (PTMs) of DRP1, with the ability to either activate or inhibit its functions based on the specific site that undergoes modification [17]. Several phosphorylation sites had been identified, including Ser579, Ser40, Ser585, Ser44, Ser592, Ser656, Ser616, Ser637, and Ser693. Among these sites, Ser-616 and Ser-637 had been extensively investigated [18]. Phosphorylation of DRP1 at Ser616 promotes mitochondrial division [19], while phosphorylation of DRP1 at Ser637 hinders mitochondrial fission [20].

Phosphorylation of Ser-616 was known to be catalyzed by several proteins, including Rho-associated protein kinase (ROCK), PKCδ, cyclin-dependent kinase 1 (CDK1), ERK1/2, and calmodulin-dependent protein kinase II (CaMKII) [11]. Recent research has shown that the ERK-DRP1 axis was activated in various diseases, including septic cardiomyopathy, malignant gliomas and pulmonary arterial hypertension [21–23]. Following phosphorylation by ERK, DRP1 translocated from the cytoplasm to the outer mitochondrial membrane, where it polymerized into a ring structure and underwent GTP-dependent constriction, culminating in mitochondrial division [24]. However, to date, it remains uncertain whether lactate-induced proliferation/migration of pulmonary fibroblasts entails Drp1-dependent mitochondrial fission, and the precise mechanisms contributing to the exacerbation of pulmonary fibrosis remain elusive.

Pulmonary fibrosis originates from an inflammatory response elicited by lung tissue damage, characterized by the aggregation and activation of inflammatory cells alongside the release of pro-inflammatory cytokines. This process entails the sustained recruitment and activation of immune cells, fostering a chronic inflammatory milieu that propels fibrotic progression [25]. Moreover, the inflammatory mediators and cytokines liberated during this cascade not only directly trigger fibroblast activation and collagen synthesis but also exert indirect effects on the composition and architecture of the extracellular matrix, culminating in pulmonary tissue fibrosis and scarring [26]. NF-κB is a transcription factor regulating the expression of various genes, including inflammatory cytokines, inflammatory mediators, cell adhesion molecules, among others. Its significance in the pathogenesis and evolution of pulmonary fibrosis is paramount and warrants meticulous consideration [27].

In this thesis, it was elucidated that lactate-induced phosphorylation of ERK and DRP1 triggered mitochondrial fission, consequently leading to an increase in ROS production. ROS was detrimental for the development of bleomycin-induced pulmonary fibrosis, as reflected through the activation of the NF-κB signaling pathway, subsequently resulting in the transcription of key factors such as IL-1β and TNF-α, which promoted fibroblast proliferation [26, 28–30]. In addition, there have been articles reporting mitochondrial ROS are required for hypoxic activation of P65 [31–33]. These findings could provide valuable insights into the development of pulmonary fibrosis and potentially uncovered targets for treating this condition.

## Results

### Lactate accumulation in pulmonary fibrosis was closely related to ROS upregulation

**Fig. 1:**
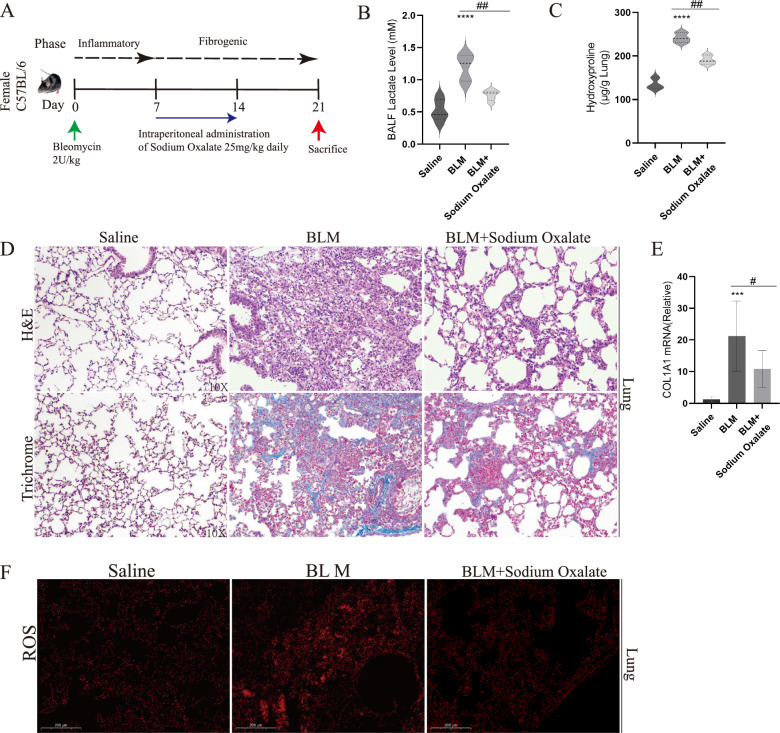
Lactate accumulation in pulmonary fibrosis was closely related to ROS upregulation. **A** Mice were administrated with Bleomycin (2 U/kg) or an equivalent volume (50 μL) of Sterile saline solution intratracheally. Daily intraperitoneal injections of sodium oxalate (25 mg/kg) were administrated on days 7 to days 14. (n = 5/group) **B** Lactate content in broncho alveolar lavage fluid (BALF) were measured with Saline, Bleomycin and Bleomycin + sodium oxalate. **C** Hydroxyproline assay of mice lung from Saline, Bleomycin and Bleomycin + sodium oxalate. **D** Lung sections from mice with Masson’s trichrome staining and H&E staining. **E** COL1A1 mRNA levels was measured by RT-qPCR. **F** ROS content was measured in lung sections. *p < 0.05, **p < 0.01, ***p < 0.001 and *p* < 0.0001, paired Student’s t-test comparing BLM group, BLM + sodium oxalate group and control group. ##*p* < 0.01, paired Student’s t-test comparing between BLM group and BLM + sodium oxalate group

Several studies had provided evidence that lactate played a significant role in the development of pulmonary fibrosis [10, 34, 35]. To verify the exacerbating role of lactate in pulmonary fibrosis, BLM-induced pulmonary fibrosis mouse model was established (Fig. 1A). In this model, sodium oxalate, a derivative of pyruvate, was added, which could inhibit lactate accumulation by blocking glycolysis [36, 37]. In mice treated with BLM, lactate concentration in bronchoalveolar lavage fluid (BALF) were reduced after treatment with sodium oxalate (Fig. 1B). Besides, hydroxyproline content in lung tissue decreased under sodium oxalate treatment (Fig. 1C). Lung structure appeared disordered according to HE staining, and collagen deposition was observed in Masson staining. However, the administration of sodium oxalate could relieve lung injury and collagen deposition (Fig. 1D). Additionally, COL1A1 mRNA decreased under sodium oxalate treatment (Fig. 1E). Meanwhile, ROS levels were increased in mice with pulmonary fibrosis, which were reduced by sodium oxalate treatment (Fig. 1F). These results indicated that the accumulation of lactate in BLM-induced pulmonary fibrosis was accompanied by a concurrent rise in ROS levels.

### ROS inhibitors ameliorated TGF-β-induced activation of fibroblasts in vitro

**Fig. 2:**
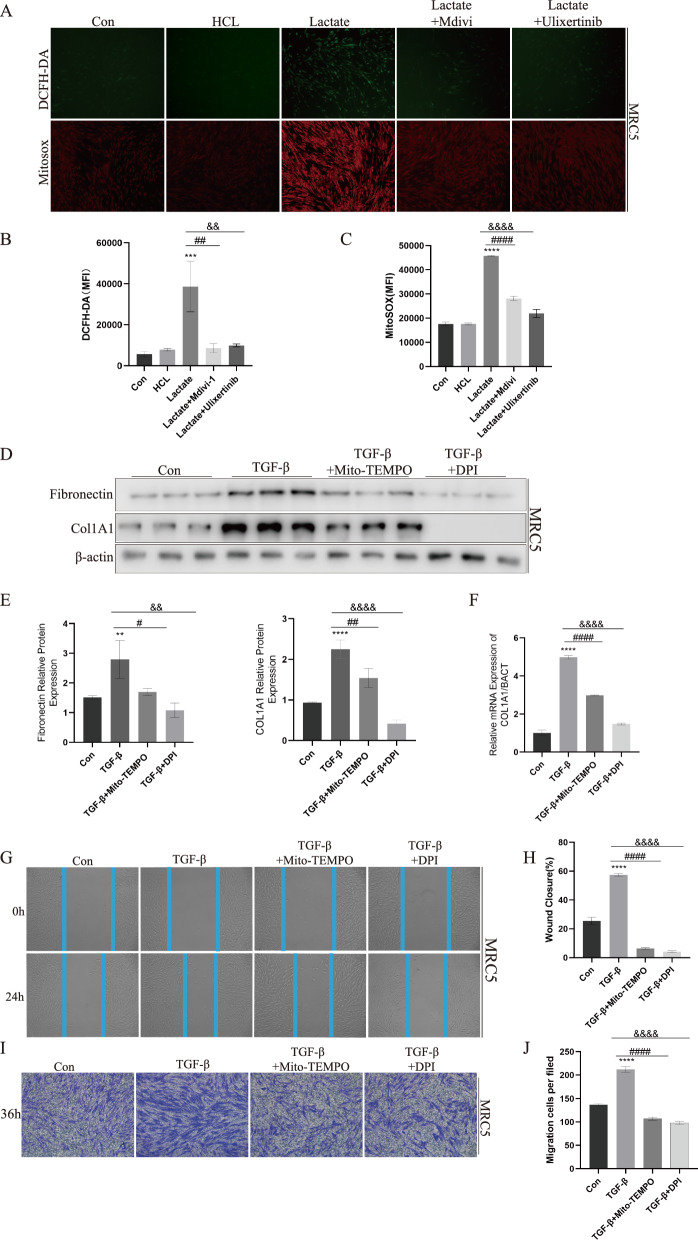
ROS inhibitors ameliorated TGF-β-induced activation of fibroblasts in vitro. **A**–**C** Lactatae induced ROS production in MRC5 cells (n = 3 per group) was determined by DCFH-DA and Mito SOX via Fluorescence microscopy and Multi Function Measuring Instrument, Mdivi-1 and Ulixertinib inhibited ROS production. **D**–**F**, The expression of COL1A1 and Fibronectin in fibroblasts increased after TGF-β stimulation, and the addition of MT and DPI could block fibroblast activation marker expression, as measured by WB and qPCR. **G**, **H** TGF-β could promote MRC5 migration which suppression by MT and DPI via Wound-Healing Assay. **I**, **J** MT and DPI could inhibit MRC5 infiltration induced by TGF-β via Transwell. **p < 0.01, ***p < 0.001, ****p < 0.0001, paired Student’s t-test comparing to control group. # *p* < 0.05, ###*p* < 0.001, ####*p* < 0.0001 comparing TGF-β group and TGF-β + MT group. &&*p* < 0.01 and &&&&*p* < 0.0001 comparing TGF-β group and TGF-β + DPI group

As a key metabolite in glycolysis, lactate had been clearly elucidated to promote pulmonary fibrosis [38]. To further explore the connection between lactate and ROS, MRC5 cells were stimulated with lactate. Lactate increased ROS as well as mitochondrial ROS levels as determined by DCFH-DA and Mito SOX staining. Importantly, treatment with HCL equivalent to lactate at the same concentration did not increase intracellular ROS levels (Fig. 2A–C). Additionally, mRNA levels of SOD1, SOD2, GPX1, GPX4 and CAT, which were main intracellular ROS scavenging related genes, were decreased 24 h after lactate stimulation of MRC5(Supplemental Figure S1). Transforming Growth Factor-β1 (TGF-β1) was a pivotal fibrogenic growth factor [39]. Hence, we employed TGF-β as a stimulus to induce the differentiation of fibroblasts. Results displayed that elevation of COL1A1 and fibronectin protein induced by TGF-β stimulation were effectively reversed in lung fibroblasts by the administration of Diphenyleneiodonium chloride (DPI) and Mito-TEMPO(MT) (Fig. 2D, E), which was mitochondrial-targeted superoxide dismutase mimetic that scavengers superoxide and alkyl radicals [40]. COL1A1 mRNA expression was also reduced by DPI and MT (Fig. 2F). Meanwhile, Primary mouse lung fibroblasts showed similar results when stimulated with DPI and MT (Supplemental Figure S2). The scratch test revealed that TGF-β stimulation enhanced wound closure, whereas DPI and MT reversed effect of TGF-β (Fig. 2G). The Transwell results also demonstrated that MT and DPI effectively inhibited fibroblast migration (Fig. 2I). The implication of these findings suggested that presence of lactate triggered production of ROS, ultimately influencing the development of pulmonary fibrosis.

### Lactate induced DRP1 activation and excessive fragmentation of the mitochondrial network in lung fibroblasts

**Fig. 3:**
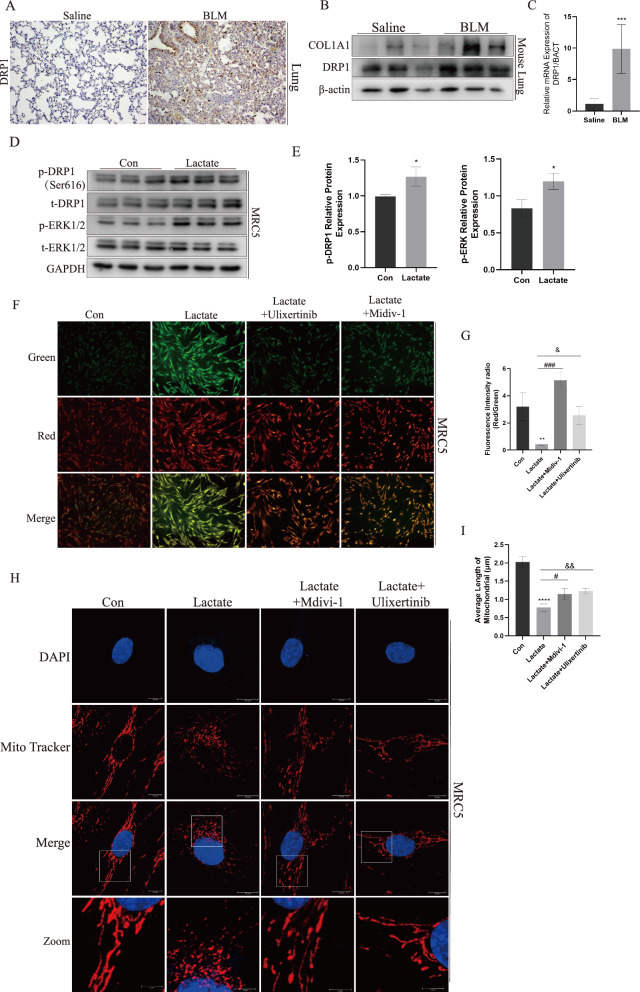
Lactate induced DRP1 activation and excessive fragmentation of the mitochondrial network in lung fibroblasts. **A** The results of IHC staining of DRP1 in saline group and BLM group. **B**, **C** DRP1 protein levels were measured in n saline group and BLM group by WB and qPCR. **D**, **E** p-DRP1S^616^ and p-ERK1/2 were detected by WB. **F**, **G** JC-1 staining in MRC5 treated with lactate, lactate + Mdivi-1 and lactate + Ulixertinib. **H**, **I** Mitochondrial network in MRC5 was analyzed by Mito Tracker staining. **p < 0.01, ***p < 0.001 and ****p < 0.0001, paired Student’s t-test comparing to control group. #*p* < 0.05 and ###*p* < 0.001, comparing lactate group and Lactate + Mdivi-1 group. &*p* < 0.05 and &&*p* < 0.01, comparing lactate group and lactate + Ulixertinib group

Excessive mitochondrial network fragmentation and alteration in mitochondrial dynamics may trigger mitochondrial dysfunction and ROS production. To validate whether there were abnormalities in the structure and function of mitochondria in pulmonary fibrosis, we examined Dynamin-related protein 1 (DRP1), a key gene responsible for regulating mitochondrial fission. The protein and mRNA levels of DRP1 displayed a significant up-regulation in BLM-induced pulmonary fibrosis in mice, as confirmed by immunohistochemistry, Western blotting, and quantitative PCR (Fig. 3A–C). To explore the relationship between lactate and mitochondrial morphological and function in lung fibrosis, we treated Human lung fibroblast cell line (MRC5) and primary mouse lung fibroblasts with lactate to explore the role of lactate in inducing mitochondrial network fission in fibroblast. The results suggested DRP1^S616^ phosphorylation was promoted by lactate treatment for 30 min (Fig. 3D, E and Supplemental Figure S3). Yet, the treatment of lactate for 12 h did not increase in DRP1 mRNA expression (Supplemental Figure S4). JC-1 staining is used to detect changes in mitochondrial membrane potential. At higher mitochondrial membrane potentials, JC-1 accumulates within the matrix of mitochondria, forming J-aggregates, generating red fluorescence. Conversely, at lower mitochondrial membrane potentials, JC-1 fails to aggregate within the matrix of mitochondria, existing instead as monomers, which generate green fluorescence [41]. The results indicated a decrease in mitochondrial membrane potential in MRC5 cells following lactate treatment. (Fig. 3F). Fluorescence intensity analysis by a multifunctional microplate reader showed similar results (Fig. 3G). Moreover, mitochondrial morphology was observed by performing confocal microscopy. Treatment with lactate resulted in increased mitochondrial fragmentation and formation of punctate mitochondria (Fig. 3H, I). These results confirmed that accumulation of lactate could impact both morphology and function of mitochondria by triggering DRP1^S616^ phosphorylation.

### p-DRP1^S616^ was activated by p-ERK1/2 and transferred to mitochondria under exogenous lactate stimulation

**Fig. 4:**
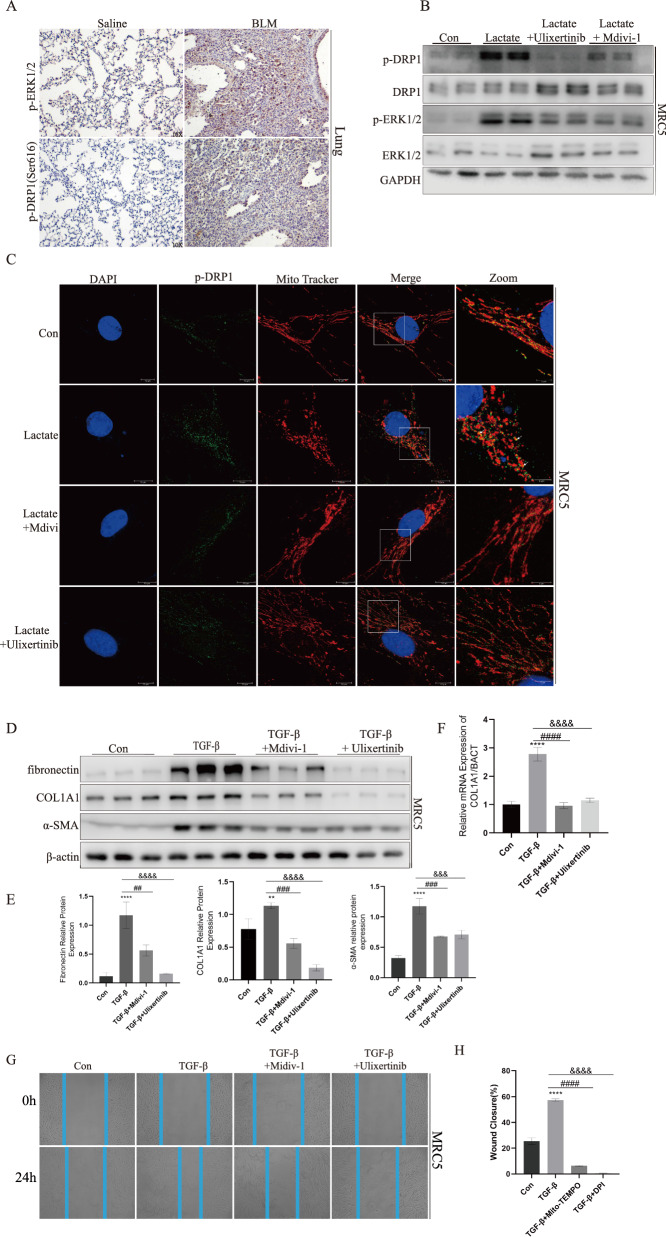
p-DRP-1^S616^ was activated by p-ERK1/2 and transferred to mitochondria under exogenous lactate stimulation. **A** p-DRP1 and p-ERK1/2 protein expression in mice with saline and BLM group was determined by IHC staining. **B** p-DRP1 and p-ERK1/2 was determined by immunoblotting with Control group, Lactate group, lactate + Ulixertinib group and lactate + Mdivi-1 group. **C** Localization of p-DRP1(green) and mitochondria (red) was determined by immunocytochemistry. **D**–**F** Fibronectin COL1A1 and α-SMA was confirmed by Western blotting and RT-qPCR with Control group, Lactate group, lactate + Mdivi-1 group and lactate + Ulixertinib group. **G**, **H** Mdivi-1 and DPI Ulixertinib could inhibit MRC5 infiltration induced by TGF-β via Transwell. **p < 0.01 and ****p < 0.0001, paired Student’s t-test comparing to control group. ##*p* < 0.01, ###*p* < 0.001 and ####*p* < 0.0001, comparing TGF-β group and TGF-β + Mdivi-1 group. &&&*p* < 0.001 and &&&&*p* < 0.0001, comparing TGF-β group and TGF-β + Ulixertinib group

Previous studies indicated that DRP1 mediated mitochondrial fragmentation was associated with ERK1/2. To determined how lactate regulated DRP1-mediated mitochondrial dynamics in fibroblast, phosphorylated ERK1/2 was detected by immunoblotting (Fig. 3D). Furthermore, in BLM-treated lung tissue immunohistochemical p-DRP1^S616^ and p-ERK1/2 staining was significantly upregulated (Fig. 4A). In this scenario, ERK1/2 kinase inhibitor Ulixertinib was used to confirm the effect of lactate on DRP1 phosphorylation. Western blot has shown that inhibition of ERK1/2 blocked lactate induced phosphorylation of Drp1^Ser−616^ (Fig. 4B). Also, lactate-induced translocation of p-DRP1 in MRC5 as determined by colocalization of p-DRP1 with Mito Tracker (Fig. 4C). To confirmed the function of Drp1 in mitochondrial network fragmentation in fibroblast, MRC5 was treated with lactate in the presence of Mdivi-1, Blocking GmPPCP-dependent DRP1 self-assembly [42]. Mdivi-1 treatment blocked lactate induced fragmentation of mitochondrial network in MRC5 (Fig. 3G). In addition, Mdivi-1 blocked lactate induced translation of DRP1 to mitochondrial (Fig. 4C). Also, MRC5s cell treated with TGF-β exhibited significantly higher level of α-SMA, COL1A1, Fibronectin expression than Control group, but Mdivi-1 and Ulixertinib could decreased expression of three markers of TGF-β-treated MRC5 cells (Fig. 4D, E). The detection of collagen expression by qPCR showed similar results to protein expression (Fig. 4F). The results obtained from qPCR and Western blotting analyses in primary mouse lung fibroblasts exhibited concordant content, reinforcing consistency of the findings (Supplemental Figure S5). The scratch assay additionally demonstrated that Mdivi-1 and Ulixertinib could diminish closure of wounds (Fig. 4G, H). These data showed that lactate had the ability to phosphorylate ERK and DRP1^S616^, which subsequently caused mitochondrial fission.

### Lactate promoted nuclear translocation of P65 through ROS and contributed to the development of pulmonary fibrosis.

To further investigate how lactate promotes fibroblast activation, we examined the status of the NF-κB signaling pathway. It is known that the transcription of P65 is necessary for the expression of fibrosis-related markers [43–45], and P65 is a transcription factor activated downstream of ROS [46].

**Fig. 5:**
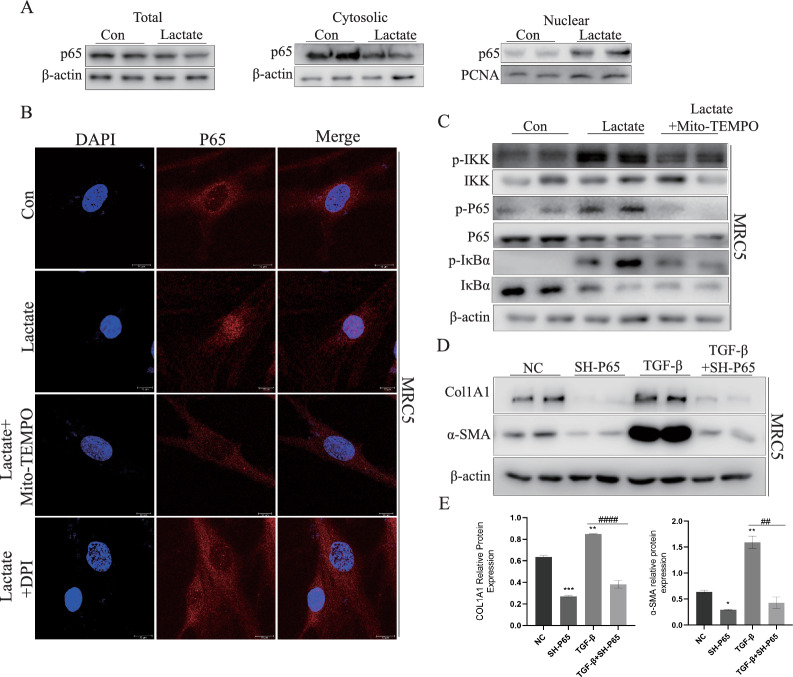
Lactate promoted nuclear translocation of P65 through ROS and contributed to the development of pulmonary fibrosis. **A** Western blotting was performed for expressions determination of p65 in total fraction, cytoplasm fraction and nucleus fraction of MRC5. **B** p65 nuclear translocation assessed by immunofluorescence staining in HMCC97H cells with control group, lactate group, lactate + MT group and lactate + DPI group. **C** phosphorylation of NK-κB signaling components in MRC5 with control group, lactate group, lactate + MT group was determined by Immunoblotting. **D**, **E** COL1A1 and α-SMA was tested by Western blotting through SH-P65. *p < 0.05, **p < 0.01 and ***p < 0.001, paired Student’s t-test comparing to control group. ##*p* < 0.01 and ####*p* < 0.0001, comparing TGF-β group and TGF-β + SH-P65 group

Therefore, following a half-hour stimulation of fibroblasts with lactate, cytoplasmic content of P65 decreased, while its nuclear content increased (Fig. 5A). Simultaneously, these findings from confocal microscopy revealed that lactate facilitated P65 translocation into nucleus. However, the effects of lactate were counteracted by MT and DPI (Fig. 5B). More specifically, Lactate promoted phosphorylation of IKK, IKBα and P65, which could be reversed by mtROS inhibitor (Fig. 5C). These results showed lactate activated P65 by increasing mtROS, which in turn caused expression of downstream related genes. Subsequently, lentivirus expression system was utilized to stably knockdown P65 in MRC5 cells, and SH-P65#1, which exhibited a more pronounced knockdown effect, was chosen for further experiments (Supplemental Figure S6). Knockdown of P65 resulted in reduced levels of COL1A1 and α-SMA proteins (Fig. 5D, E). These charts showed that ROS could promote translocation of P65 into nucleus, thereby regulating expression of downstream genes and contributing to exacerbation of pulmonary fibrosis pathogenesis ultimately.

### Inhibitors targeting ERK1/2, DRP1, and mtROS exhibited alleviated BLM-induced mouse pulmonary fibrosis

**Fig. 6:**
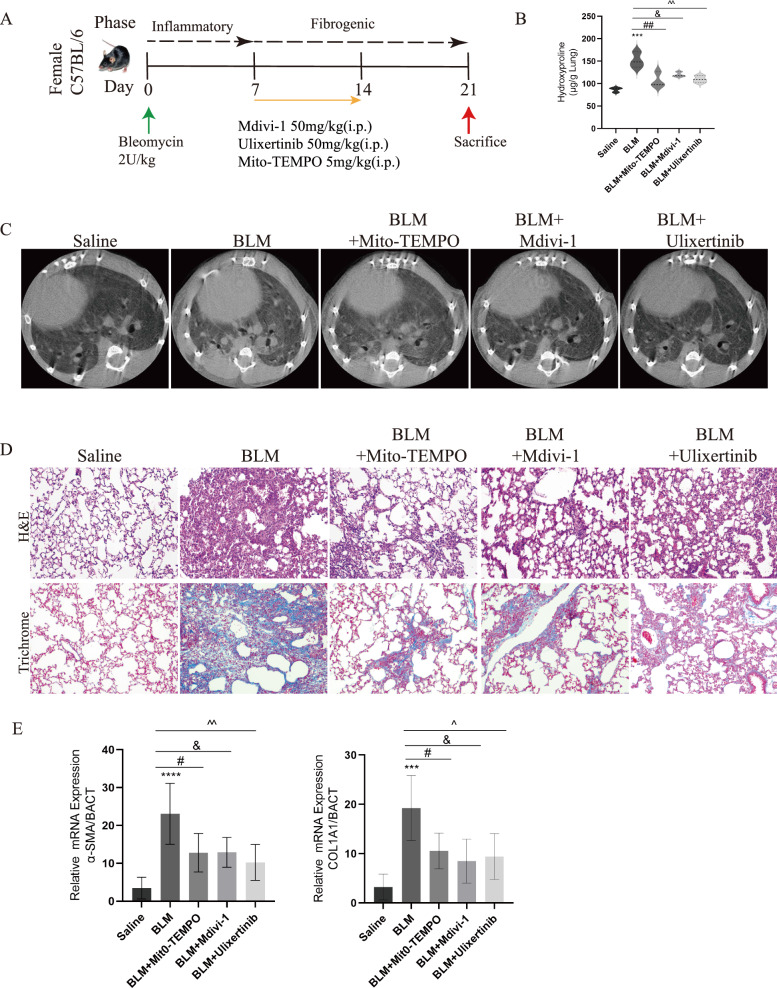
Inhibitors targeting ERK1/2, DRP1, and mtROS exhibited alleviated BLM-induced mouse pulmonary fibrosis. **A** The construction of BLM-induced pulmonary fibrosis mouse models for Mito-TEMPO, Mdivi-1 and Ulixertinib. **B** Mito-TEMPO, Mdivi-1 and Ulixertinib blunts established fibrosis in mouse model. **C** Different groups with radiographic features as determined by micro-computed tomography (micro-ct), healthy lungs are black and diseased lungs are increasingly white (elevated density). **D** Masson's trichrome staining an H&E staining were performed on representative lung sections (n = 3) obtained from each experimental group of mice. **E** qPCR results showed changes in COL1A1 and α-SMA mRMA expression after inhibitor treatment. ***p < 0.001, paired Student’s t-test comparing to control group. #*p* < 0.05 and ##*p* < 0.01, comparing TGF-β group and TGF-β + MT group. &*p* < 0.05, comparing TGF-β group and TGF-β + Mdivi-1 group. ^*p* < 0.05 and ^^*p* < 0.01, comparing TGF-β group and TGF-β + Ulixertinib group

To explore the effect of abnormal mitochondrial fission on BLM-induced fibrosis in mice, we intraperitoneally injected Ulixertinib, Mdivi-1, Mito-TEMPO, respectively, one week after BLM administration (Fig. 6A). The treatment with the three inhibitors, Ulixertinib, Mdivi-1, and Mito-TEMPO, significantly reduced the hydroxyproline content in fibrotic mice lungs. (Fig. 6B). The Micro-CT imaging results indicated an increase in localized lung density within BLM group, signifying an expansion of the parenchymal area (manifested by an increased presence of white areas in the images), while treatment with Ulixertinib, Mdivi-1, and Mito-TEMPO reduced lung fibrotic regions (Fig. 6C). Pathological staining results indicated that Ulixertinib, Mdivi-1, Mito-TEMPO ameliorated Bleomycin-induced lung structural disorders and collagen deposition (Fig. 6D). Expression of COL1A1and α-SMA mRNA was reduced after inhibitors treatment (Fig. 6E). These dates demonstrated inhibition of ERK-DRP1-mediated mitochondrial fission ameliorated BLM-induced pulmonary fibrosis.

## Discussion

IPF was a progressive, irreversible interstitial lung disease with complex pathogenic factors, high mortality, and currently no effective treatment [47]. Whenever IPF occurs, it was commonly accompanied by lactate accumulation and production of reactive oxygen species (ROS) [48, 49]. The relationship between these two factors has been a subject of great interest and discussion among researchers [50, 51]. This article focused primarily on the changes of fibroblasts in a pulmonary fibrosis model. The research indicated that lactate promoted the activation of fibroblasts as well as the generation of ROS. However, different cell types played an important role in pulmonary fibrosis, and further exploration of other cell types was also urgently needed.

Lactate is generated from pyruvate, the end product of glycolysis, catalyzed by LDHA, thereby regulating intracellular pH and sustaining energy metabolism [52]. Targeted LDHA inhibitors, such as Sodium Oxamate and Gossypol, have been extensively reported in numerous studies and have shown significant efficacy in animal models of various diseases [53–56]. These research findings suggested that targeting the LDHA-mediated pathway offered a novel approach to combat pulmonary fibrosis and halt disease progression. Previous research demonstrated that the glycolysis inhibitor 2-deoxy-d-glucose (2-DG) could decrease lactate levels in fibrotic lungs and significantly improved bleomycin-induced pulmonary fibrosis in mice. These findings highlighted other metabolites also played crucial roles in disease progression. Additionally, our team had been focusing on the roles of lactate and glycolytic intermediates in disease. We planned to continue investigating this issue in our future studies. Furthermore, recognizing the complexity of IPF pathogenesis is essential, as targeting a single enzyme or pathway may not be sufficient to fully address the underlying disease pathology. Although lactate dehydrogenase inhibitors show promise in preclinical studies, careful evaluation of drug safety and efficacy remains essential.

It has been reported that inhibiting MCT4 expression resulted in an increase in intracellular lactate concentration and levels of ROS. Metabolic processes occurring within mitochondria were widely recognized as major contributors to production of ROS, which showed a well-established function in cell signaling [57]. This study demonstrated that lactate promoted mitochondrial fission, leading to an increase in mitochondrial ROS levels. However, the roles of intracellular and extracellular lactate in inducing ROS production required further investigation. And targeting lactate emerged as a potential therapeutic strategy for pulmonary fibrosis in forthcoming interventions.

Mdivi-1 is a small molecule inhibitor targeting DRP1, which has been demonstrated to inhibit apoptosis in ischemic retinal cells and chondrocytes [58, 59]. The ERK1/2 inhibitor Ulixertinib demonstrates anti-proliferative efficacy in Pediatric Low-Grade Gliomas (pLGG) and holds substantial clinical potential [60]. This study demonstrated that lactate activated ERK1/2, resulted in the phosphorylation of DRP1^S616^, which enhanced mitochondrial abnormal fission and disrupted mitochondrial membrane potential, leading to mtROS elevation. The interventions with Mdivi-1 and Ulixertinib had reversed adverse process, alleviating pulmonary fibrosis. GPR81 is a specific receptor for lactate, and its activation inhibited adenylate cyclase activity and cAMP formation. Importantly, cAMP has been reported to inhibit RAF1/MEK/ERK signaling [61]. This might be one of the reasons why lactate promoted ERK1/2 phosphorylation. Nevertheless, it required further investigation to determine whether lactate could induce DRP1^S616^ phosphorylation in pulmonary fibrosis through other mechanisms.

Numerous studies had shown that under oxidative stress conditions, excessive ROS could destroy cellular proteins, lipids and DNA, leading to fatal cell damage, which was involved in a variety of pathologies, such as aging, cancer, neurodegenerative diseases, cardiovascular diseases and diabetes, among others [62–65]. Physiological role of ROS is mainly based on its ability to regulate multiple signaling pathways, including NF-κB, MAPK, p53, Keap1-Nrf2 and PI3K/AKT [66]. Therefore, the mechanism of ROS affecting pulmonary fibrosis needs to be further explored.

Mitochondrial ROS (mtROS) production was increased by a high mitochondrial transmembrane potential (ΔΨ), which acted as an energy barrier for electron transport [67]. Mito-TEMPO was a mitochondria-targeted superoxide dismutase mimic and antioxidant. In mammalian systems, Mito-TEMPO effectively mitigated oxidative stress-induced mitochondrial structural abnormalities by scavenging mitochondria-derived superoxide [68]. This article mainly investigated the role of lactate in exacerbation of pulmonary fibrosis by inducing mitochondrial ROS production. Mito-TEMPO treatment inhibited TGF-β-induced fibroblast differentiation and attenuated bleomycin-induced pulmonary fibrosis. Yet, the article did not address whether ROS from other sources were also affected by lactate. For example, NOX4, the major enzymatic source of ROS production by myofibroblasts and NOX2, which plays an vital role in the innate immune response [69, 70]. Therefore, the relationship between lactate and ROS from other sources needs to be further investigated.

In the process of pulmonary fibrosis, the P65 component within the NF-κB complex played a significant role, with its activity and regulation affecting the occurrence and development of pathological processes such as inflammation and fibrosis [71]. Nevertheless, the influence of P65 extends beyond boundaries of immune responses, encompassing transcriptional regulation that impacts cell survival, differentiation, and proliferation [72]. In this study, it was found that lactate caused P65 translocation to nucleus, thereby influencing expression of various P65 downstream genes. These genes included hypoxia-inducible factors, inflammation-related genes, and pro-vascular endothelial growth factors [29, 73], which respond to lactate induction need further validation. Treatment with MT, a specific inhibitor of mitochondrial ROS, inhibited P65 activation while also ameliorated pulmonary fibrosis. Hence, considering ERK-DRP1 aix and mitochondrial ROS as targets could serve as a therapeutic approach for pulmonary fibrosis.

## Conclusion

Overall, lactate contributed to mitochondrial fragmentation through ERK1/2-DRP1 axis, leading to ROS generation and subsequent P65 activation, which further aggravated pulmonary fibrosis. Administering inhibitors of ERK1/2 and DRP1^S616^ phosphorylation resulted in a decrease in generation of ROS. Moreover, use of Mdivi-1, Ulixertinib, and Mito-TEMPO successfully reversed pulmonary fibrosis induced by BLM in mice. These findings suggested that the involvement of ERK1/2-DRP1 in mitochondrial fission and generation of ROS were crucial steps in the progression of pulmonary fibrosis. This understanding of the mechanism opens up new possibilities for the treatment of idiopathic pulmonary fibrosis.

## Materials and methods

### Reagent and antibody

Lactate was obtained from Sigma (St. Louis, MO, USA), Antibody against DRP1(#8570), p-DRP1^S616^(#3455), p-IκB-alpha(#2859S), p-P65(#3033S) and P65(#8242) were purchased from Cell Signaling Technology (Boston, MO, USA). Antibody against fibronectin (#15,613–1-AP) and COL1A1(67,288–1-Ig) were purchased from Proteintech (Wuhan, China). Antibody against IκB(#AF5002), IKK(#AF6014), p-IKK(AF3013), ERK(#AF0155), p-ERK(#AF1015), β-Actin(#AF7018) and GAPDH(#AF7021) were purchased from Affinity (Changzhou, China).

### Cell culture

Human embryonic lung fibroblast MRC5 cell line was purchased the Type Culture Collection of the China Academy of Sciences, Shanghai, China. MRC5 was maintained at 37 ℃ in MEM supplemented with 10% fetal bovine serum and 100 U/ml penicillin and 100 μg/ml streptomycin in a humidified atmosphere of 5% CO_2_. Cells were used up to the fifth passage and were seeded onto different types of plates for further experiments once they reached approximately 75% of the desired cell density.

Fibrotic model was created in vitro by utilizing 10 ng/mL TGF-β (Novoprotein, Shanghai, China). The cells were pretreated with 10 mM lactate, 10 μM Mdivi-1, 20 μM Ulixertinib, 20 nM Mito-TEMPO and 10 μM DPI, all of which purchased from MedchemExpression ((New Jersey, USA).

### Mouse primary lung fibroblast extraction

All animal experiments in this manuscript were approved by the Biology Academic Committee of Hehe Normal University.

Mouse lungs were removed under aseptic conditions and placed in Hanks solution, and lung tissue was minced and digested with 0.25% trypsin for 40 min. The cell suspension was centrifuged at 1500 r/min for 5 min to allow for sedimentation. The resulting precipitate was resuspended in DMEM medium for a heavy suspension, followed by centrifugation at 800 r/min for 5 min. Afterward, the supernatant was further centrifuged at 1500 r/min for 5 min, resulting in purified lung fibroblasts.

### Quantitative real-time PCR

Total RNA was extracted from tissues and cells using TRIzol reagent in accordance with the manufacturer's instructions. The extracted RNA samples were then reverse transcribed into complementary DNA (cDNA) using the HiScript II Reverse Transcriptase (Vazyme, Nanjing, China). The expression of genes of interest was quantified using qPCR with Hieff qPCR SYBR Green Master Mix (Yeasen Biotechnology, Shanghai, China). The housekeeping gene ACTB was used as an internal control. The relative mRNA expression levels were determined using the Livak method. The primers used for RT-qPCR were synthesized by Sangon Biotech. The primers required for qPCR were shown in Table 1.

**Table 1.** Primer Sequence

Gene	Sence (5'–3')	Anti-sense (5'–3')
Human-COL1A1	GAGGGCCAAGACGAAGACATC	CAGATCACGTCATCGCACAAC
Mouse-COL1A1	GCTCCTCTTAGGGGCCACT	ATTGGGGACCCTTAGGCCAT
Human-SOD1	CTCACTCTCAGGAGACCATTGC	CCACAAGCCAAACGACTTCCAG
Human-SOD2	CTGGACAAACCTCAGCCCTAAC	AACCTGAGCCTTGGACACCAAC
Human-GPX1	GTGCTCGGCTTCCCGTGCAAC	CTCGAAGAGCATGAAGTTGGGC
Human-GPX4	ACAAGAACGGCTGCGTGGTGAA	GCCACACACTTGTGGAGCTAGA
Human-CAT	GTGCGGAGATTCAACACTGCCA	CGGCAATGTTCTCACACAGACG
Human-DNM1L	GATGCCATAGTTGAAGTGGTGAC	CCACAAGCATCAGCAAAGTCTGG
Mouse-DNM1L	GCFGAACCTTAGAATCTGTGGACC	CAGGCACAAATAAAGCAGGACGG
Mouse-ACTA2	GTCCCAGACATCAGGGAGTAA	TCGGATACTTCAGCGTCAGGA

### Immunofluorescence

The cells were attached to glass coverslips by using 4% paraformaldehyde, rinsed with PBS, and subsequently permeabilized with 0.1% Triton X-100 for 10 min. Samples at room temperature were treated with h 5% bovine serum albumin in PBS to block non-specific binding for 1 h. Cells were incubated with anti-p-DRP1(S616) for overnight 4 ℃. The specific secondary antibody, conjugated with GFP fluorochrome (Invitrogen, Carlsbad, CA), was incubated at room temperature for 1 h in the dark. After PBS washes, the nuclei were stained with DAPI. Images were captured using an optical microscope.

### Determination of mitochondrial network fragmentation

Cultivate MRC5 cells on coverslips in a 24-well plate, followed by overnight incubation. Upon cell adhesion, treat the cells with lactate, Mdivi-1, and Ulixertinib. Subsequently, stain the cells with the mitochondria-specific fluorescent probe MitoTracker Deep Red (100 nM, purchased from Beyotime) for 30 min. Conduct a 10-min treatment of MRC5 cells with 0.1% Triton X-100, followed by DAPI restaining to label cell nuclei. Finally, fix the coverslips onto glass slides and capture images using confocal microscopy. Perform quantitative analysis of the mitochondrial network using the MiNA plugin in ImageJ.

### Western blotting

Proteins were obtained from tissue and cells using RIPA buffer and concentration were measured by a bicinchoninic acid assay Kit. Protein lysates, ranging from 20 to 50 μg, were separated on SDS–polyacrylamide gels with a concentration of 10 to 15%. After electrophoresis, the proteins were transferred to polyvinylidene difluroride membranes (Millipore, Billerica, MA, USA), and then blocked with 5% BSA at room temperature for 1 h. The membranes were incubated overnight at 4 °C with specific primary antibodies previously described. β-actin was used as the internal control. The membranes were then rinsed with TBST buffer and treated with HRP-conjugated secondary antibodies for 2 h at room temperature. After three washes of TBST buffer, protein bands were visualized Odyssey® XF Imaging System.

### H&E staining

Ex vivo fresh lung tissue was fixed with 4% paraformaldehyde for 24 h at room temperature, then embedded in paraffin and cut into 5 μm sections by a rotary microtome. The dried sections were deparaffinized, rehydrated, and then stained with hematoxylin and eosin. The images were captured using a light microscope. Scores were assessed by a pathologist on the basis of the integrity of lung tissue, alveoli, and the extent of mononuclear infiltration.

### Masson’s trichrome staining

After deparaffinization and rehydration, sections were stained with Weigert iron hematoxylin staining solution for 5–10 min. After washing with water, sections were stained with Ponceau fuchsin staining solution for 5–10 min. Finally, sections were stained with aniline blue staining solution for 1–2 min.

### Immunohistochemistry

Paraffin sections were stained with antibodies against DRP1, p-DRP1, p-ERK1/2 and passed overnight at 4 ℃. After incubation with the secondary antibody, it was detected by the diamine benzidine method. After the nuclei were stained with hematoxylin, the images were presented by light microscopy.

### Total ROS and mitochondrial ROS (mtROS) detection

Intracellular ROS levels were measured by incubation with DCFH-DA (Beyotime, shanghai, China) at 37 °C for 30 min. mtROS were detected after incubation with mitoSOX Red (Thermofisher, USA) at 37 °C for 30 min.

### Cell transfection

For the stable P65 knockdown cell line, the p65 shRNA fragment was cloned into PLKO.1, and then transfected into 293 T cells together with pmd2G and pspax2 to package the complete virus. Finally, the virus was used to infect normal cells.

### Statistical analysis

All data were analyzed and presented as graphs using Graphpad Prim 8 (GraphPad Software, La Jolla, CA, USA). The data were presented as mean ± standard deviation (SD). All the data met the assumption of normal distribution. For comparisons between two groups, we used the two-tailed Student's t-test. Comparisons among three or more groups were conducted using one-way analysis of variance (ANOVA) followed by Tukey's test. A p-value less than 0.05 was considered statistically significant.

### Supplementary Information

Additional file 1 (PDF 428 KB)

## Data Availability

The authors confirm that the data supporting the findings of this study are available within the article and its supplementary materials. The data that support the findings of this study are available from the corresponding author, GY, upon reasonable request. No additional resources were generated for this study.
