# Supplementary material for: Lactate facilitated mitochondrial fission-derived ROS to promote pulmonary fibrosis via ERK/DRP-1 signaling
Source: J Transl Med. 2024 May 21;22:479. doi: 10.1186/s12967-024-05289-2 (PMC11106888; doi:10.1186/s12967-024-05289-2)
Supplement: Supplementary file 1 — Additional file 1 (PDF 428 KB) [file 12967_2024_5289_MOESM1_ESM.pdf]

Supplemental

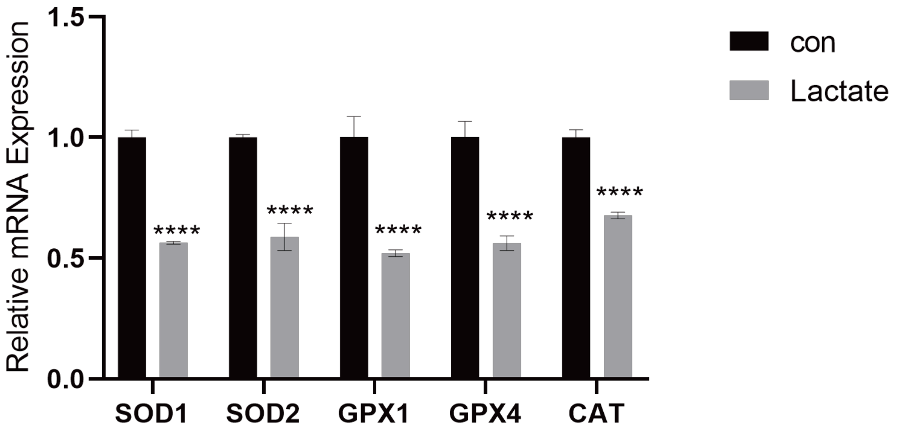

Figure S1. SOD1, SOD2 GPX1, GPX4 and CAT were measures by RT-qPCR after lactate treatment 24h.

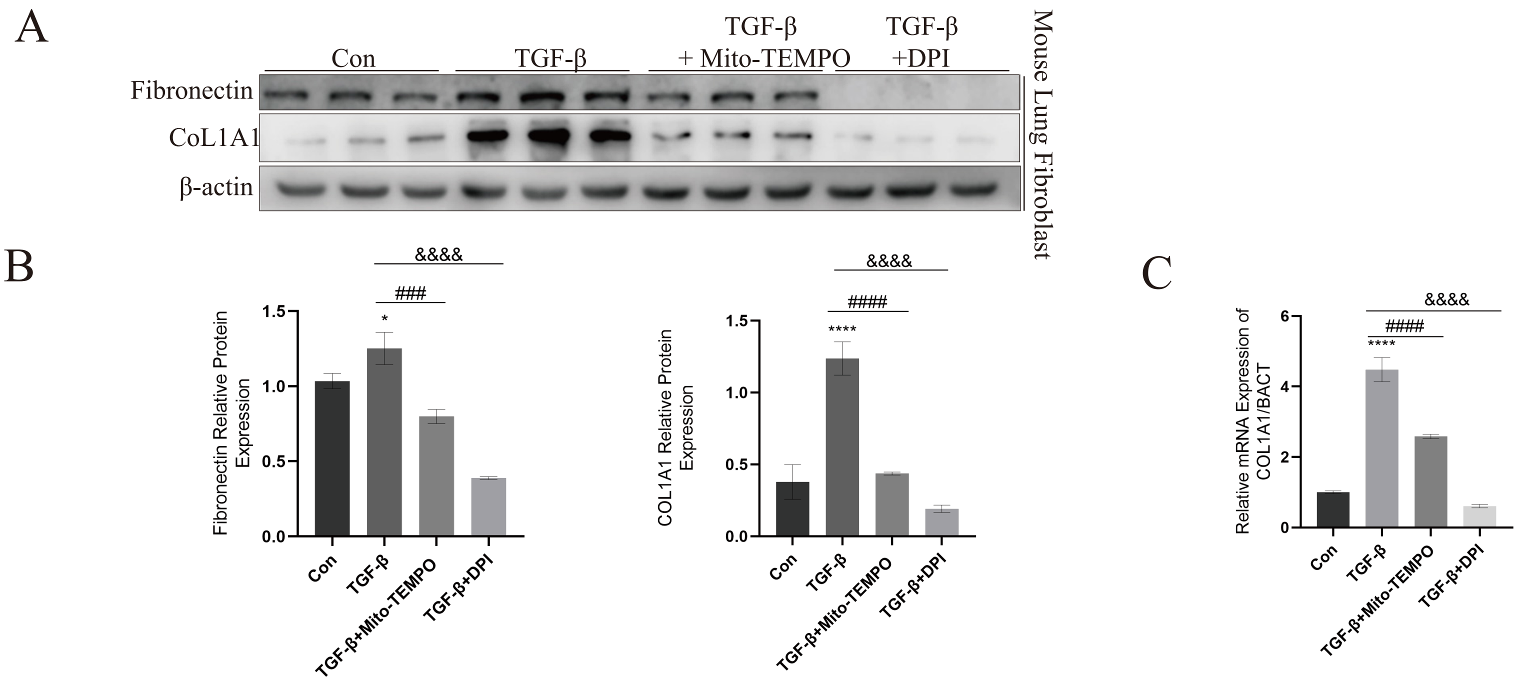

Figure S2. （A-B）Fibronectin and COL1A1 were detected in primary mouse lung fibroblasts by WB with control group, TGF-β group, TGF-β+ MT group and TGF-β+DPI group.

（C）Relative mRNA of COL1A1 in primary mouse lung fibroblasts was detected.

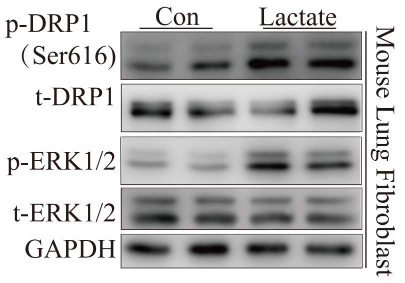

Figure S3 Primary mouse lung fibroblasts were treated lactate for 30 minutes, p-DRP1S616 and p-ERK1/2 was determined by WB.

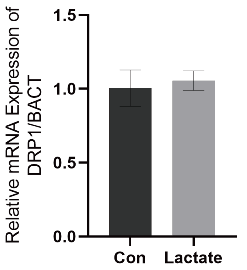

Figure S4 The expression of DRP1 mRNA in MRCS treated with lactate for 12h was determined

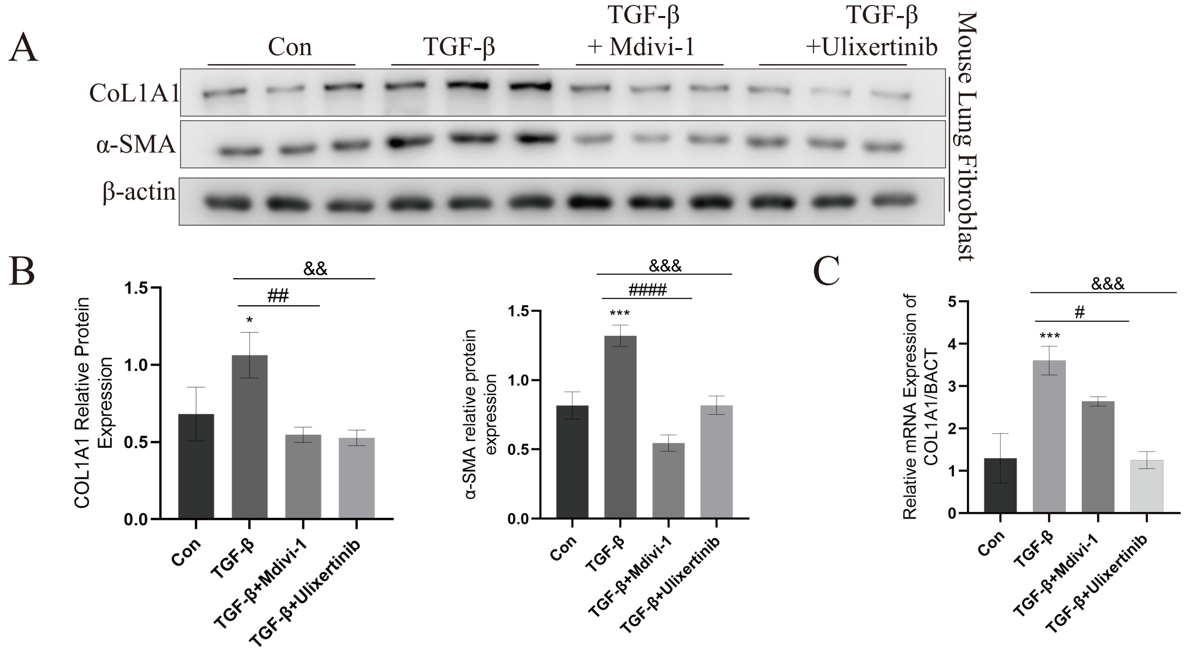

Figure S5. （A-B）α-SMA and COL1A1 were detected in primary mouse lung fibroblasts by WB with control group, TGF-β group, TGF-β+ Mdivi-1 group and TGF-β+ Ulixertinib group.

(C) Relative mRNA of COL1A1 in primary mouse lung fibroblasts was detected.

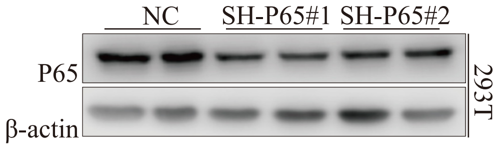

Figure S6. Western blotting results showed the effect of P65 knockdown in 293T cells.
